# Properties of 3D Printing Fiber-Reinforced Geopolymers Based on Interlayer Bonding and Anisotropy

**DOI:** 10.3390/ma15228032

**Published:** 2022-11-14

**Authors:** Chun Lv, Hongtao Shen, Jie Liu, Dan Wu, Enxiang Qu, Shuang Liu

**Affiliations:** 1 College of Architecture and Civil Engineering, Qiqihar University, Qiqihar 161006, China; 2 Zhongdingruizhi Construction Development Co., Ltd., Qiqihar 161005, China; 3 College of Light-Industry and Textile Engineering, Qiqihar University, Qiqihar 161006, China; 4 Engineering Research Center for Hemp and Product in Cold Region of Ministry of Education, Qiqihar 161006, China

**Keywords:** fiber, 3D printing, geopolymer, anisotropy, interlayer bonding, workability, mechanical property

## Abstract

The engineering applications and related researches of 3D printing fiber-reinforced geopolymers are becoming more and more extensive. However, compared with traditional mould-casted cement-based materials, the properties of 3D-printed fiber-reinforced geopolymers are significantly different, and their interlayer bonding and anisotropy effects are less studied, so in-depth analysis and summary are needed. Similar to common cement-based materials, the reinforcement fibers for geopolymers include not only traditional fibers, such as steel fibers and carbon fibers, but also synthetic polymer fibers and natural polymer fibers. These fibers have unique properties, most of which have good mechanical properties and bonding properties with geopolymers, as well as excellent crack resistance and enhancement. This paper summarizes and analyzes the effects of traditional fibers, polymer fibers, plant fibers and other reinforcement fibers on the properties of 3D-printed fiber-reinforced geopolymers, especially on the interlayer bonding and anisotropy. The influence of the flow and thixotropic properties of fiber-reinforced fresh geopolymer on the weak bond and anisotropy between layers is summarized and analyzed. At the same time, the influence of fibers on the compressive strength, flexural strength and interlayer binding strength of the hardened geopolymers is investigated. The effect of fibers on the anisotropy of 3D-printed geopolymers and the methods to improve the interlayer binding degree are summarized. The limitations of 3D printing fiber-reinforced geopolymers are pointed out and some suggestions for improvement are put forward. Finally, the research on 3D printing fiber-reinforced geopolymers is summarized. This paper provides a reference for further improving the interlayer bonding strength of 3D-printed fiber-reinforced geopolymers. At the same time, the anisotropy properties of 3D-printed fiber-reinforced geopolymers are used to provide a basis for engineering applications.

## 1. Introduction

Based on the concept of green construction, 3D printing technology is more and more widely used in construction engineering. Compared with traditional manufacturing, 3D printing technology has less impact on the environment and requires less energy. In recent years, more and more engineering applications and related studies have been conducted to improve the properties of cement-based and geopolymers using fiber reinforcement [1,2,3]. These reinforcement fibers include not only traditional inorganic fibers [4,5,6], but also synthetic polymer fibers [7,8,9,10,11] and natural fibers [12,13,14]. At the same time, these fibers are also gradually used for the 3D printing of fly ash (FA) cement-based composites and geopolymers (3DPGs) [15,16]. As we all know, the core principle of 3D printing technology is layer-by-layer manufacturing, and the forming processes mainly include D-Shape process and Contour Crafting [17,18,19]. Unlike the traditional process, the 3DPGs molding process does not require the installation of templates, which has the advantages of the fine process and rapid molding [20,21,22]. 3DPGs fresh slurry has continuous and uniform extrudability, good cohesiveness between printing layers, and appropriate setting time, which meet the free molding of slurry [23,24]. It is challenging to set steel bar when 3DPGs are printed and formed, so fibers are added to enhance the strength and toughness of 3DPGs [25], forming a new type of 3D printing fiber-reinforced FA-cement-based composites or geopolymers (3DFRPGs). The main drawback of 3DPGs is their weak adhesion between printing layers. In the extrusion printing process, the shrinkage and evaporation of printing wire caused by excessive surface water promote the decrease in5 interlayer adhesion. The mechanical properties, bonding properties and durability of hardened composites are damaged by it.

As a green building material, geopolymer has the advantages of high early strength, fast hardening speed, and a wide range of raw materials, which can replace cement-based materials as 3D printing materials in the future [26,27,28]. The research and application of geopolymers as 3D printing materials have attracted more and more attention from scientists. Geopolymer is a kind of inorganic polymer material, which is the mixed reaction of active low-calcium aluminum silicon material and alkaline activator. Under the curing condition of less than 150 °C, a new type of inorganic aluminum silicon cementitious material with spatial three-dimensional network bond structure similar to organic polymer is prepared. It has a three-dimensional network structure composed of [SiO_4_] and [AlO_4_] tetrahedral elements. Geopolymer is an environmentally friendly cementing material with low energy consumption and less pollutant emission in the production process [29,30].

The synthesis of geopolymer requires two materials: active solid silicone–aluminate and an alkaline solution containing alkali metals and silicates or aluminates. The alkaline solution plays the binder, alkali activator, and dispersant [31,32]. The concept of geopolymers was introduced by the French scientist Davidovits in 1978. He stimulated geological minerals with alkali metal silicate solution under strongly alkaline conditions to form polymeric aluminum silicate materials [33]. Subsequently, other solid silicate materials, such as FA, volcanic ash, and ground granulated blast furnace slag (GGBS), were used to produce geopolymers successfully [34,35]. Because geopolymer is ceramic, its flexural and tensile strength are poor, and it is sensitive to microcracks. To solve the brittleness problem of geopolymers, the toughness of composites can be improved by adding fibers [36,37]. Adding fibers to geopolymers can limit the growth of cracks and enhance the ductility, toughness, and tensile strength of geopolymers [38,39,40]. The summarization table of previous findings of 3DPGs on filler materials and its related analysis as additive materials can be seen in Table 1. Table 1 shows the fiber types and the composition of 3DPGs. The data in the table are converted according to the percentage of cementitious materials.

The binders of 3DPGs are mainly geopolymers, including FA, GGBS, silicon (SF) powder, and metakaolin. Sometimes cement is added. Fine aggregate is generally fine sand, and the amount added is roughly equal to the binders. To improve the properties of geopolymers, admixtures are sometimes added. At the same time, researchers also added fibers and other functional materials to prepare different types of 3DFRPGs composites. Since fiber type and content, fiber length, and aspect ratio can affect the performance of 3DPGs, the performance of 3DPGs will be significantly different when different types of fibers are selected.

In recent years, many studies on 3DFRPGs mainly focus on the single fibers and their mechanical properties. There is a lack of comparative studies on multiple fiber 3DPGs, especially on the interlayer binding and anisotropy of 3DFRPGs. In this paper, the effects of different fibers on the properties of fresh and hardened 3DFRPGs are compared and analyzed. The factors affecting the interlayer binding and anisotropy of 3DFRPGs are studied, and improvement measures are proposed. From the perspective of affecting the workability of fresh 3DFRPGs mixtures, the effects of different types of fibers on the interlayer bonding and anisotropy of 3DPGs are summarized, and the mechanical properties of 3DFRPGs after hardening are also reviewed. At the same time, the interlayer binding strength and anisotropy of 3DFRPGs are analyzed. Finally, the conclusion and prospect of 3DFRPGs are given. This paper provides a reference for further improving the interlayer bonding strength of 3DFRPGs. At the same time, the anisotropy property of 3DFRPGs is used to provide a basis for engineering applications. In the following, the classification and characteristics of reinforcement fibers, polymerization of geopolymers, properties of fresh and hardened 3DFRPGs, interlayer bonding strength and anisotropy, and improved methods of soft surfaces between 3DFRPGs layers are analyzed.

## 2. Reinforced Fibers of 3DPGs

**Figure 1:**
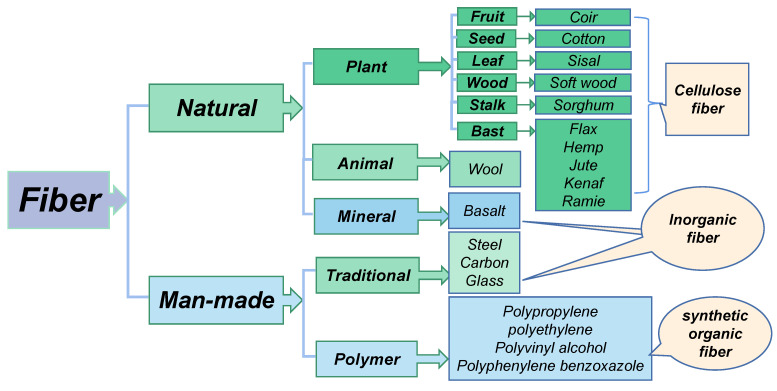
Classification of reinforced fibers of geopolymers.

Generally, the fibers added in the 3D-printed geopolymer slurry can be divided into natural and man-made fibers from their sources [51]. Natural fibers mainly include natural plant fibers (PFs), natural animal fibers, and natural mineral fibers, and man-made fibers mainly include synthetic organic fibers and part of the traditional inorganic fibers. The traditional inorganic fibers include steel fibers, carbon fibers, glass fibers, and basalt fibers. In recent years, there have been many kinds of synthetic fibers and PFs that are widely used. The classification of reinforced fibers commonly used in 3D printing cement-based materials and geopolymers is shown in Figure 1.

The mechanical properties and geometric characteristics of different types of fibers are varied, which can affect the performance of 3DPGs. The comparison of properties of commonly used fibers is shown in Table 2. The synthetic organic fibers have similar mechanical properties with high tensile strength and tensile modulus as carbon fibers. There are many kinds of PFs. Many PFs have relatively high strength and specific strength. They are a kind of green materials that meet the requirements of sustainable development.

It can be seen from Table 1 and Table 2 that the volume fraction of 3DFRPGs fiber content is 0.25–2.0%, and the fiber length is 2–20 mm. The research on the influence of fiber on the performance of 3DPGs mainly focuses on traditional fiber, synthetic fiber, natural PF, and so on. Among synthetic fibers, polypropylene (PP), polyethylene (PE), and polyvinyl alcohol (PVA) fibers have been studied more on the material properties of 3D printing mortar. The main components and property measurements of 3DFRPGs in some studies are shown in Table 3.

As can be seen from Table 3, the activators are mainly NaOH + K_2_SiO_3_, K_2_SiO_3_ or Na_2_SiO_3_. The property measurements include the performance of fresh and hardened 3DFRPGs. The reinforced fibers used include glass fibers, steel fibers, and polymer fibers. Fiber contents are 0.5–2.0%. The content of FA is more than 50% in the binders, which is the most important component. FA can be used alone or mixed with GGBS and SF as precursor materials for geopolymers. The measured properties are mainly related to the thixotropy of fresh 3DFRPGs, and the compressive and flexural strength of the 3DFRPGs after hardening. The research on interlayer bonding and anisotropy is relatively little. Therefore, it is necessary to vigorously promote the study of these characteristics of 3DFRPGs.

## 3. Polymerization of Geopolymers

In general, cement-based composites have poor high-temperature resistance and corrosion resistance, and geopolymers have well overcome this shortcoming [62]. The main components of geopolymer are precursor and activator. The precursors include alkali silicate glass cementitious materials and alkali silicate mineral cementitious materials. The alkali silicate glass cementitious materials take amorphous silicate glass as raw materials, such GGBS and FA, while the alkali silicate mineral cementitious materials include natural minerals, such kaolin and clay [63]. The activator is an alkaline solution of alkali metals and silicate or aluminate.

As previously described, the alkaline activator dissolves silicon and aluminum in the precursor and catalyzes the condensation reaction with them. The response of aluminosilicate oxide in a strong basic activator solution breaks the Si-O-Si bond, and subsequently, Al atoms penetrate the original Si-O-Si structure. The resulting aluminosilicate oxide gel based on the Si-O-Al block is the precursor of the polycondensation reaction. The aluminosilicate gel phase is a highly reactive product. Under alkaline conditions, rapid chemical reactions form rigid 3D polymers and a ring frame of Si-O-Al bonds [64].

According to Duxson et al. [65], the polymerization of geopolymers can be divided into four reaction stages: dissolution, diffusion, polymerization, and solidification. Specifically, under the action of a strong alkali solution, the Si-O bond and Al-O bond of the precursor active material break, forming a series of oligo-Si-O tetrahedra and Al-O tetrahedra similar to polymer monomer. Under the action of strong alkali, these oligo-Si-O tetrahedra and Al-O tetrahedra are gradually dehydrated and polymerized, thus forming geopolymers with three-dimensional network structures [66].

Geopolymers are composed of fibers and matrix with different properties, and the interface is formed by the contact surface of fibers and matrix. The interface of composite is a very complex microstructure, which includes the geometric surface and transition region of matrix and fibers. Usually, the properties of the interfacial layer between fiber and matrix can be optimized by adjusting the interfacial bonding state to achieve the best performance of geopolymer composites.

## 4. Effect of Fibers on Properties of Fresh 3DFRPGs Mixture

As mentioned above, the strength of anisotropy of 3DPGs is closely related to the workability of the freshly mixed slurry. All the infill patterns have a directional dependency. The printing interface between the layers is a key factor affecting the structure’s performance [67]. 3DPGs are formed layer by layer, which is easy to cause weak bond between layers, resulting in anisotropic behavior.

### 4.1. Formation of Weak Interlaminar Bonding and Anisotropy

In geopolymer slurry deposition, the weak bonding surface between the printed layers becomes more evident due to the accumulation of material weight without external vibration [60]. To some extent, the fragile bonding surface between the printed layers becomes the potential defect of the printing structure, which leads to the inconsistent deformation and discontinuous mechanical properties of the structure, resulting in anisotropy [55,59]. In addition, the predetermined plane or path propagated by the crack also produces anisotropic behavior. The bearing capacity values of different directions of loading are different. Component rupture occurs due to local stress concentration, which weakens the overall bearing capacity and durability of the structure.

Due to the requirements of the printing process, in addition to the fluidity, the thixotropy and setting time should also be controlled, which are the key factors affecting the continuous and uniform extrusion of mortar and ensuring the pouring molding. In addition to selecting the appropriate binder, fine aggregate, admixture, and other necessary materials, adding fiber materials in the slurry can restrict the deformation of the structure, prevent the spalling of the printing slurry, and obtain a uniform and continuous printing effect.

### 4.2. Effects of Inorganic Fibers

Traditional inorganic fibers are commonly used in 3DFRPGs. Yalçınkaya [68] prepared three layers of printed steel fiber-reinforced cement-based specimens to study the effect of hydroxypropyl methylcellulose dosage on the mechanical properties of 3DFRPGs. The measured porosity of the three-layer printing specimen was 3.9–4.7% higher than that of the single-layer printing specimen. The permeability test results were consistent, and the permeability of the three-layer specimen was higher than that of the single-layer specimen. In addition, the increase in hydroxypropyl methylcellulose and fiber reinforcement resulted in slightly higher porosity values. Such narrow pores primarily occur between the printed layer interfaces. Such pores can be observed around steel fibers and are mainly composed of encapsulated voids and macropores. The local porosity between layers depends on the surface roughness after extrusion, the initial yield stress, the thixotropy of the mixture, the time interval between layers, the hardness of the printed layer, and the surface moisture. Ma et al. [69] printed short carbon fiber-reinforced geopolymer to study the rheological properties of fresh 3DFRPG mixtures. The apparent viscosity, thixotropy, storage modulus, loss modulus, and yield stress of modified and unmodified geopolymer inks with different contents of short carbon fibers were compared. It was found that the addition of fibers facilitated the extrusion of the mixture from the micronozzle, maintaining the filamentous shape and supporting subsequent printing layers. Archez et al. [70] used 3D printing technology to shape a hollow cylinder using geopolymers and additives. They added a small amount of wollastonite, glass fibers, or inactive aluminum silicate to the mixture and adjusted the printing speed to realize the printing of composites. 3DFRPG printing layers have good adhesion, avoiding the cold joint effect, and the fibers show flow orientation during stacking. Zhong et al. [71] analyzed 3DPGs of glass fibers. The bending deflection of the composites improved with the increase in fiber content. When the fiber content increased from 0.25 to 1.0%, the bending ductility in the vertical direction of the printing layer increased by 16.67 to 135.29%, which could be attributed to the fiber–matrix interaction after the crack initiation. With the increase in fiber content, the number of effective fibers will increase, which can resist crack propagation. The bridge fiber will slip during the pulling-out process, so the ductility of the entire composite will enhance. The effect of glass fiber length is less sensitive than that of glass fiber content.

Panda et al. [60] used short glass fiber reinforcement and F-grade fly ash to prepare the 3DFRPGs mixture. The test results were consistent with Ma et al. [52]. Due to the effect of interlayer deposition, when the fiber content increased to 1%, it had apparent direction dependence. The printing process deposits directional filamentous printing layers, resulting in layered structure and mechanical anisotropy. In addition, the non-hydrophilicity of most inorganic fibers leads to a slightly poor bond with the matrix, and its dosage should not be too high. Otherwise, it is unfavorable to the fluidity of the mixture. In general, the fiber content of 3DFRPGs is limited to 1% to avoid clogging the nozzle and to achieve smooth, continuous extrusion during printing.

### 4.3. Effects of PFs

PF is a kind of natural fiber. PF is widely used in nature, rich in resources, has good properties, and has broad development prospects in fiber-reinforced geopolymers.

Kong et al. [72] proposed new 3DFRPGs based on industrial and agricultural waste slag such as FA, GGBS, kenaf straw core and kenaf fiber. To evaluate the extrusion and printing properties of different geopolymers, the viscosity evolution, shape retention, and cross-section characteristics of different geopolymers were studied. The results showed that compared with the control group, the viscosity recovery rate and thickness shape retention rate of the 3DFRPGs mixture supplemented with kenaf rod powder and kenaf fiber increased by 189.19% and 67.63%, respectively. The dry density decreased from 1749.32 kg/m^3^ to 1560.60 kg/m^3^, which is very conducive to multilayer printing. In addition, the skeletal action of the kenaf rod and the bridging action of kenaf fiber in geopolymer were confirmed by SEM at the microscopic level. The rheological analysis of 3DFRPGs by Long et al. [73] showed that the plastic viscosity of 3DFRPGs mixture supplemented with 1 wt% micro-crystalline cellulose was increased by 20.9% compared with that of the mix without micro-crystalline cellulose. The workability of the mix containing 1.0 wt% of micro-crystalline cellulose was also improved. The printing structure has neither large cracks nor deformation of the printing layer during the printing process. Sinka et al. [74] used agricultural waste cannabis tablets to prepare 3DFRPGs. The results show that the biocomposite with flax strip and fast-setting binder has shape stability and constructability.

From the existing literature, there are relatively few studies on the use of PFs as reinforcements for 3DFRPGs. In practice, the compatibility of PFs and geopolymer is good and the bonding property between PFs, and matrix is suitable, which needs further study in the future [40].

### 4.4. Effects of Synthetic Organic Fibers

Synthetic organic fibers are abundant, and excellent in performance. In 3DFRPGs, PP fibers, PVA fibers and PE fibers are widely used. Ye et al. [75] studied the fluidity effect of fresh 3DFRPGs mixture under different PE fiber content. It was found that the spread diameter and penetration depth of the fresh mixture decreased with the increase in fiber content, and the fluidity became worse. The expanded diameter of the fresh 3DFRPGs mixture with 2.0% PE fiber content was 144 mm, which decreased by 14.8% compared with the mixture with 1.0% PE fiber content. Zhu et al. [76] added 1%, 1.5%, and 2% volume fractions of PE fibers to strengthen the 3DFRPGs matrix, respectively. The properties of the prepared fresh 3DFRPGs mixture, including workability, rheological property and constructability, were experimentally investigated. Different from steel fibers, the fluidity of the mixture with polyvinyl fibers became worse [38].

Similar to PE fiber, increasing the content of PP fiber can also reduce the fluidity of the mixture. Nematollahi et al. [77] used PP fibers to adjust the fluidity of fresh mortars. The fluidity of fresh mortars could be improved by adding carboxymethyl cellulose to the mixture. The test showed that the effect of decreasing fiber content on the fluidity of fresh mortar was greater than that of increasing carboxymethyl cellulose content.

When PVA fibers are added to a fresh 3DFRPGs mixture, the fluidity of the new 3DFRPGs mixture decreases significantly with the increase in fiber content. The results showed that 1–1.4% PVA fiber content could ensure the fluidity of fresh 3DFRPGs mixture was controlled within 170–180 mm, and this is an excellent value of fluidity. With the increase in the content of PVA fibers, the setting time difference of fresh 3DFRPGs mixture was not more than 6 min, indicating that the range of fibers had no significant effect on the setting time of fresh 3DFRPGs mixture [78,79].

Malaszkiewicz et al. [80] used polymer fibers with a volume fraction of 0–4% to conduct experiments on the fluidity and setting time of fresh mortar. It was found that the yield strength of mortar was not significantly affected by different polymer fiber types and lengths. Over time, the fluidity loss slowed compared to the mixture without fiber. The setting time of the fresh 3DFRPGs mixture developed by Le et al. [81] was up to 100 min. At the same time, experiments also showed that adding polymer fibers could effectively improve the thixotropic properties of fresh 3DFRPGs mixtures [82].

Different types of fibers have little effect on the properties of fresh 3DFRPGs mixture. The fluidity of the mixture decreases when other types of fibers are added. Due to the water absorption properties of PFs, this is more evident in the 3DPGs mixtures reinforced by PFs and some synthetic polymer fibers. In fact, the reduced fluidity of the fresh 3DFRPGs mixture is not a negative effect because printing requires both high thixotropy, and the incorporation of plant and polymer fibers allows for better buildability of the fresh 3DFRPGs mixture.

## 5. Effect of Fibers on Mechanical Properties of Hardened 3DFRPGs

**Figure 2:**
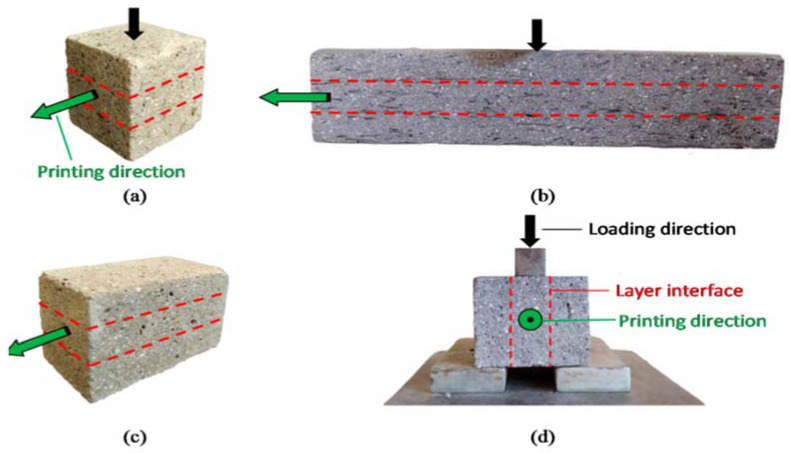
The specimen preparation for mechanical tests [68]. (**a**) Compressive specimen (35 × 35 × 40 mm); (**b**) flexural specimen (35 × 40 × 160 mm^3^); (**c**) shear specimen (35 ×40 × 60 mm); (**d**) specimen of interlaminar bonding strength (35 × 40 × 60 mm^3^). Reproduced with permission from Yalçınkaya, Buildings; published by MDPI, 2022.

The mechanical properties of hardened 3DFRPGs mainly include compressive strength, tensile strength, flexural strength, shear strength, and other indexes. The mechanical properties of 3DFRPGs include not only the composites themselves but also the interfacial bonding strength between adjacent printing layers. Yalçınkaya et al. designed mechanical properties test specimens [68] for each index using three-layer printing 3DFRPGs specimens, as shown in Figure 2.

This figure shows the specimen form commonly used in 3DFRPGs mechanical property tests. Figure 2a is the compressive strength specimen, Figure 2b is the flexural strength specimen, and Figure 2c is the shear strength specimen; Figure 2d is the interlaminar bonding strength specimen. It can be seen from the figure that the composites exhibit noticeable anisotropy due to different printing orientations leading to different fiber orientations in the matrix. Different loading directions are selected for the test of the mechanical properties of composite materials, and the test results are also different.

### 5.1. Effects of Inorganic Fibers

The effect of fiber on the mechanical properties of 3DPGs is noticeable, especially on flexural strength. Ma et al. [69] studied the mechanical properties of short carbon fibers reinforced 3DPGs. In 3DFRPG composites, the uniform orientation distribution of short carbon fibers mainly enhanced the mechanical properties. When the fiber content was 3 wt %, the flexural strength and compressive strength of 3DFRPGs were 309.2% and 375.8% higher than those of pure 3DPGs, respectively. Due to the hierarchical ordered structure and complex interface, 3DFRPGs exhibit superior bearing capacity and ductile failure mode. The dense lamellar structure and mechanical anisotropy between the printing layers significantly enhance the fracture resistance of 3DFRPGs, but the crack orientation is insensitive. Archez et al. [70] designed a hollow cylinder with glass fibers and successfully printed 3DFRPGs with a bending strength of 15 MPa. Ma et al. [69] also conducted experimental research on the mechanical behavior of basalt fiber-reinforced 3DPGs under compressive, tensile, flexural, shear loads and obtained good results.

Ma et al. [44] printed 3DPGs specimens configured with three different printing paths. The composition of 3DPGs specimens was fly ash 64 wt %, silica fume 11 wt %, slag 25 wt %, silica sand, sodium metasilicate pentahydrate powder, water, micro steel cable and a small amount of polypropylene fiber 0.56 wt %. In addition, appropriate viscosity modification admixture increased its water retention properties, thus avoiding bleeding. The extruder continuously fed the micro steel cable into the printing nozzle through the conveying tube and then simultaneously extruded the micro cable and deposited filaments to form the micro steel cable reinforced composite. All specimens were composed of eight layers of filaments (12 mm), with a micro steel cable (1.2 mm) at the center of each filament. Three unstiffened geopolymer specimens were prepared for comparison. The three specimens include FPA (Skew cross Filament), FPB and FPC (Orthogonal cross filament).

3DFRPGs have obvious ductility and post-peak toughness. On the contrary, the non-enhanced geopolymers showed significant brittleness. 3DFRPGs can still bear a considerable increase in deflection under the condition of load reduction, indicating that the post-cracking bending moment capacity is enhanced. However, when the non-fiber reinforced structure reaches the flexural strength, the load decreases obviously, and the flexural deflection is low. Three different printing paths have a particular influence on the mechanical bending resistance. The flexural strength of 3DFRPGs is 5.1, 5.6, and 2.1 times that of the non-reinforced composites in paths A, B, and C, respectively. The buckling strength of FPA was the highest, followed by FPB and FPC specimens. It is shown that the skewing cross filament (FPA) can withstand tensile stress better than the orthogonal cross filament (FPB, FPC), because the filament perpendicular to the tensile stress contributes less to its mechanical ability. The failure and crack patterns of 3DPGs with and without micro cable are significantly different. The multi-crack failure of 3DFRPGs indicates the synergistic effect of the micro cable and geopolymer matrix on the applied bending load. The stress borne by the fibers can be well transmitted to the matrix, and both jointly respond to deformation and cracks. In contrast, a single main crack was observed in the non-reinforced specimen, similar to the conventional brittle materials.

Due to the effect of interlayer deposition, when the fiber content increased to 1%, the fiber alignment direction was consistent with the bending direction [47], and there was apparent direction dependence. The tensile strength of specimens with loading direction perpendicular to the printing layer and loading direction parallel to the printing layer showed an upward trend. For perpendicular loading, the good bond strength between layers usually exhibited good tensile strength. Under parallel loading, fiber distribution was the key factor in determining the strength. Due to the lower bond strength, higher tensile strength was obtained under parallel loading than in the perpendicular direction. In addition to the fiber distribution, the fiber/matrix interface bond was very significant in improving the performance of 3DFRPGs. The porosity of the matrix is reduced when the appropriate amount of fiber is present in the matrix.

### 5.2. Effects of PFs

**Figure 3:**
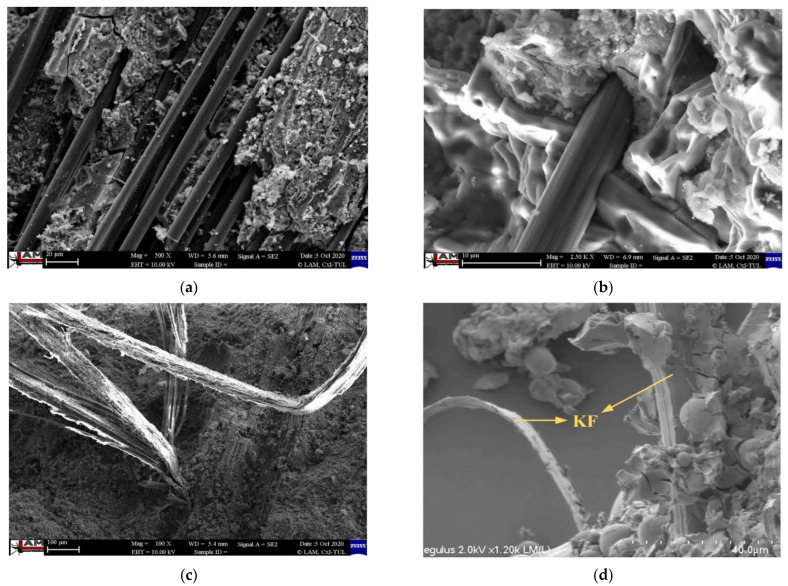
Microstructures of 3DFRPGs with different reinforcements. (**a**,**b**) Carbon fibers; (**c**) flax fibers [83]; Reproduced with permission from Korniejenko et al., Int. J. Mol. Sci.; published by MDPI, 2022. (**d**) Kenaf fibers [72]. Reproduced with permission from Kong et al., Constr. Build. Mater.; published by Elsevier, 2022.

Compared with traditional inorganic fibers, PFs have different bonding properties with geopolymers. It can be seen from Figure 3 that the microstructure of carbon fiber-reinforced 3DPGs is significantly different from that of flax or kenaf fibers, as shown in Figure 3b–d. Although carbon fibers are uniformly distributed in the matrix, there is still single fiber aggregation, as shown in Figure 3a [83]. For carbon fiber-reinforced 3DPGs, the gap between fibers and matrix is visible. There is an apparent lack of bond between the fibers and the matrix. The study did not confirm the cohesion of carbon fibers and geopolymer matrix, which may be the main reason why the mechanical properties of carbon fiber-reinforced 3DPGs are not as good as those of flax fiber-reinforced 3DPGs. Kong et al. [72] added 0.2% kenaf fibers to 3DPGs, and the kenaf fibers bonded closely with the matrix and did not break even after mechanical destruction, showing an internal bridging effect. Kenaf stalks, and kenaf fibers had significant differences in the bending strength of 3DPGs. The bending strength of 3DPGs doped with 0.2 wt % kenaf fibers increases, and the increased amplitude varies with the type of extrusion nozzle. The compressive test showed that the compressive strength of 3DPGs three-layer printing sample with 0.2 wt % kenaf fiber could reach 8.39 MPa after curing for 28 days. Long et al. [73] found that the yield stress of mortars supplemented with 1 wt % microcrystalline cellulose was 190.0% higher than that of mortars without microcrystalline cellulose. Compared with mortar without micro-crystalline cellulose, the 28-day compressive strength and flexural strength of the mortars with 1 wt % of microcrystalline cellulose increased by 18.6% and 12.5%, respectively. In recent years, 3DFRPGs can be prepared from waste materials such as hemp shives [74], which also have shape stability and buildability. Of course, compared with inorganic fibers, the research on the application of PFs in 3DFRPGs is relatively little, and needs to be increased in the future.

### 5.3. Effects of Synthetic Organic Fibers

Synthetic organic fiber is a kind of polymer fiber. Synthetic organic fibers commonly used include PE fibers, PP fibers, and PVA fibers. Synthetic organic fibers have excellent properties and are mostly used in 3DFRPGs.

#### 5.3.1. Effect of PE Fibers

Both steel fibers and synthetic organic fibers have the properties of controlling cracking and increasing toughness after reaching the peak load. PE fiber-reinforced geopolymers can avoid the sudden fracture of steel fiber-reinforced geopolymers in the later loading stage [38]. The application performance of synthetic organic fiber-reinforced geopolymer is higher than that of steel fiber-reinforced geopolymer.

Ye et al. [75] showed that compared with 3DFRPGs without fiber, 3DFRPGs with 1.0% PE fiber content had a slightly higher tensile strain capacity. The bending strength and energy dissipation ability of 3DFRPGs with 1.5% PE fiber content are the best. Apparent anisotropy was observed in compressive strength and flexural energy dissipation capacity. Zhu et al. [76] used PE fibers with different volume fractions to test the compressive, bending, and uniaxial tensile properties of 3DFRPGs. It has good strain-hardening behavior, with a tensile strength of 5.7 MPa and a tensile strain capacity of 11.4%.

#### 5.3.2. Effect of PP Fibers

The effect of PP fibers on the mechanical properties of 3DFRPGs is similar to that of cement-based materials. Nematollahi et al. [41] prepared 3DPGs by adding 0.25, 0.5, 0.75, and 1.00 vol % PP fibers to the mixture, respectively. The compressive strength of 3DPG without PP fibers was 22 MPa, while the compressive strength of 3DPG with 0.25% PP fibers was increased to about 36 MPa. The results showed that the fibers aligned parallel to the extrusion direction helped to form crack bridging under the action of vertical pressure. After fiber incorporation, the failure mode of 3DFRPGs changes from brittle to ductile, and its properties depend on fiber contents. 3DFRPGs with a fiber content of 0.25% and a fiber content 0.5% showed flexural softening. However, 3DFRPGs with a fiber content of 0.75% and 1% showed flexural hardening behavior. The low fiber contents have no significant effect on the bending strength of 3DPGs compared with the specimens without and with 0.25 vol % fibers. The increase in fiber contents will lead to a rise in matrix porosity. At the same time, the higher the fiber contents, the stronger the fracture bridging ability and the higher the flexural strength, thus offsetting the negative effect of the high porosity. In the case of high 1.0 vol % fiber content, the fiber fracture bridging effect is greater than the negative effect of porosity.

#### 5.3.3. Effect of PVA Fibers

Paul et al. [82] prepared 3DFRPGs using PVA fibers. The penetration strength was 0.05 MPa at the initial printing stage and 0.7 MPa after 30 min. The compressive strength for 28 days is 40 MPa. The experimental results showed that the PVA fibers could effectively improve the compressive strength of 3DFRPGs, and its 1-day compressive strength could reach 50% of the 28-day compressive strength. The flexural strength of 3DFRPGs increased rapidly with the increase in PVA fiber content, indicating that PVA fiber plays an influential bridging role in 3DFRPGs matrix. On the other hand, the PVA fibers were oriented along the printing path, which significantly improved the flexural strength of 3DFRPGs. Liu et al. [84] found that PVA fibers significantly improved the anti-shrinkage property of 3DFRPGs, and their cracking index and drying shrinkage decreased by 73.85–100% and 9.73–16.22%, respectively. The maximum flexural strength of 3DFRPGs was 72.43% higher than that of cast concrete. At the same time, the total porosity decreased by 31.08%, which optimized the pore structure of the matrix. The mechanical properties of the matrix are improved. Therefore, the alignment effect of PVA fibers can effectively improve the mechanical properties and anti-shrinkage properties of the matrix [85].

### 5.4. Effects of Hybrid Fibers

3DFRPGs with single fibers can improve the matrix properties, but there are some relative shortcomings. Therefore, the scientists mixed fibers with different types. Thus, 3DFRPGs with better performance can be obtained. Tosun-Felekoglu et al. [86] found that the influence of hybrid fibers on the mechanical properties of the mixture was similar to that of single fibers through bending experiments of PP fiber- and PVA fiber-reinforced composites. With the increase in hybrid fiber content, the deformation ability of the composite was further improved, and the number of cracks in the matrix was significantly reduced. Kong et al. [72] used kenaf stalks and kenaf fibers to strengthen 3DPGs, which effectively improved the structure and performance of the matrix. The mechanical properties of multi-fiber reinforced 3DPGs were tested. It was found that polymer fibers and inorganic fibers were used together, and the effect was good. Le et al. [81] used PP and alkaline glass fibers to improve the crack resistance of 3DPGs. Lim [57] mixed long steel wires with short PVA fibers and enhanced the flexural strength of 3DFRPGs by 290%. Similar to the single fibers, the compressive strength of 3DFRPGs did not change significantly after adding hybrid fibers. When the fiber content reached 1% of the volume content, the tensile strength and flexural strength of 3DFRPGs were increased considerably. Sanjayan et al. [87] reduced the water–binder ratio and optimized the material gradation so that the compressive strength of the hardened 3DFRPGs reached 100 MPa, and the flexural strength reached 11 MPa at the same time. The performance of 3DFRPGs can be further improved by mixing hybrid fibers, incredibly soft and hard fibers, long and short fibers and other fibers with different properties into the 3DPGs matrix.

## 6. Interlayer Bonding and Anisotropy of 3DFRPGs

Due to the unique construction technology of 3D printing stack forming, weak interfaces are often formed between adjacent printing layers. The bonding performance between layers has become a key factor affecting the mechanical properties of printing specimens. The construction technology of layering and stacking leads to weak bonding and anisotropic mechanical properties between layers.

### 6.1. Interlayer Bonding Strength of 3DFRPGs

The interlayer bonding strength of fresh 3DFRPGs mixture is closely related to the fiber content. The interlayer bonding strength of the PP fiber-reinforced 3DPGs mixture studied by Nematollahi et al. [41] is 1.8–3.1 MPa. The interlayer bonding strength of 3DPGs containing 0.25 vol % fibers was 19% higher than the average interlayer bonding strength of 3DPGs without fibers. All 3DFRPGs with fiber content higher than 0.25 vol % had lower interlayer bonding strength than 3DPGs without fibers and containing 0.25 vol % fibers. Le et al. [81] also tested the interlayer bonding strength of PP fiber-reinforced cement-based materials, which was 2.3 MPa and decreased with the increase in interlayer printing time interval. Liu et al. [88] measured that the interlayer bonding strength of glass fiber-reinforced 3DPGs was significantly lower than that of non-fiber 3DPGs. Ye et al. [89] estimated that the interlayer bonding strength of the print specimen of PE fiber-reinforced cement-based material was 1.36 MPa.

In addition to fiber content, interlayer printing time interval, and slurry surface dehydration, the compressive strength, tensile strength, and interlayer bonding strength of 3DFRPGs are all affected. If the printing time between adjacent layers of 3DFRPGs is short, the effect of layer orientation is very minimal. With increasing the printing time interval between adjacent layers, the interlayer binding strength will decrease. Wolfs et al. [90] studied the effect of interlayer interval time and surface dehydration on compressive and tensile strength. It was found that the impact of layer orientation was very minimal for sufficiently short interlayer intervals. However, the interlayer bonding strength decreased with the increase in interlayer interval time. The decrease in intensity was more pronounced for specimens exposed to dry conditions at intervals, indicating that the interlayer bonding strength of the 3DFRPGs mixture was dependent on the setting time and the water content of the interlaminar interface [91]. Chaves et al. [92] tested 3DFRPGs with different print layers. The number of layers of 3DFRPGs had a significant influence on the bending hardening response. The number of cracks with more than one printed layer was low, and the deflection value at the maximum bending strength was also common, indicating that the ductility of the 3DFRPGs with more printing layers decreases. There are two main reasons for this behavior. The first one is purely geometric, resulting in higher tensile stresses in the tensile zone due to the increase in the moment of inertia of the section. As a result, the fiber is pulled out more rapidly to absorb tensile stress, limiting crack propagation and flexural strength recovery. On the other hand, it indicates that the interface bonding force is weak.

Concrete materials are similar to geopolymers. For fiber-reinforced concrete materials, the type of fiber has a crucial influence on the interlayer bonding properties. Sanjayan et al. [87] made a double-layer rectangular test block, pre-cut a 5 mm incision between layers, and measured that the bonding strength between layers was only 0.65 MPa through a direct tensile test. Zareiyan et al. [93] enhanced interlayer bonding by setting bumps between printing layers to form a self-locking effect. Marchment et al. [94] strengthened the interlayer bonding by inserting steel reinforcement. Li et al. [95] set raised ribs between layers of PE fiber-reinforced 3DPGs to improve the interlayer bonding performance of 3DFRPGs.

In general, the interlayer bonding strength of 3DPGs is reduced by fiber incorporation, especially for some inorganic fibers. The reason is that the stiffness of some inorganic fibers, such as glass fiber, is more significant. Due to the limitation of nozzle size, the orientation distribution occurs during the printing process, and the orientation is parallel to the interlayer interface. This kind of rigid fiber is difficult to realize directional distribution in the printing process and cannot bridge the indirect interface of the printing layer. However, flexible fibers can form Bridges between layers, improving adhesion between layers. On the other hand, after adding fibers, the fluidity of the slurry is reduced, and the adhesion between the printing layers is relatively weakened, which further reduces the binding effect between each printing layer.

### 6.2. Anisotropy of 3DFRPGs

Due to the layered printing, there are macroscale pores on the slurry interface, and the interface binding ability is weakened. The properties of the slurry also have significant performance differences in different directions of space, with apparent anisotropy, and the compressive strength and elastic modulus in a specific direction are low [96,97]. On the other hand, during the flow of the printed slurry, the fibers tend to separate and float, resulting in different fiber distributions and isotropic orientations in the structure.

The mechanism of the influence of different fibers on the anisotropy of 3DPGs matrix is complex, which can be explored by mechanical tests in several directions. Ding et al. [98] studied the anisotropic behavior of 3DFRPGs under bending. After the addition of PE fibers, the failure of the specimen was no longer dominated by the weak interface, the flexural strength of the three directions was significantly improved. The post-peak performance was directly related to the fiber content. The microstructure analysis showed that the uniform arrangement of fiber orientation was the critical factor leading to the improvement of ultimate strength and post-peak performance. In the printing plane, the flexural strength in the direction parallel to and perpendicular to the printing element was the highest and similar, while the flexural strength in the direction perpendicular to the printing plane was the lowest. 3DFRPGs shows apparent anisotropic behavior. Panda et al. studied the influence of different loading directions on the performance of specimens through loading tests [47]. T1, T2, and T3 were different loading directions. T1 indicated that the loading direction was perpendicular to the printing layer, and T2 indicated that the loading direction was parallel to the printing direction. T3 showed that the loading direction was perpendicular to the printing direction. When the loading direction was perpendicular to the printing layer, specimen deformation was minimized. In contrast, when the loading direction was perpendicular to the printing direction, the flexural strength was the minimum. Directional dependence is considered to be an inherent property of layered manufacturing processes [98,99]. Among T1, T2, and T3, the flexural strength of T1 is the highest, followed by T2 and T3. Due to the low interlaminar binding strength of 3DFRPGs, the deformation in the T3 direction is the smallest.

The addition of fibers decreased the compressive strength of 3DFRPGs. Under the same fiber percentage, the compressive strength of 3DFRPGs was the highest in the T3 direction, followed by T1 and T2 directions. Because the horizontal and vertical planes are composed of multiple print layers, there is little difference between T1 and T2 directions. Meanwhile, the tensile strength increases with the increase in fiber content.

In order to clarify the influence of PVA fibers on 3DFRPGs, Aslani et al. [46] prepared specimens with 1.75 vol % PVA fiber content by traditional mould casting method and compared them with 3DFRPGs. To replace FA, 5% waste crumb rubber (CR) was selected. The results of printed specimens confirm the dominant effect of anisotropy on material properties, which is evident in the bending behavior of printed elements. At 28 days, the peak stress of 3DFRPGs samples in T3 and T1 directions increased by 7% and 4%, respectively. Compared with the T1 direction, the 7-day and 28-day compressive strength of samples loaded in the T3 direction increased by 9% and 3%, respectively. This means that the loading in the parallel fiber direction produced better compressive strength than the loading in the vertical fiber direction. The 28-day flexural strength of the specimens loaded in the T1 direction increased the flexural strength of the specimens at 7 days and 28 days by 110% and 43%, respectively, compared with the 3D specimens in the T2 direction. At the age of 28 days, the flexural strength of the specimens loaded at T1 and T2 was 153% and 76% higher than that of the mould-casted specimens, respectively. The specimens of 3DFRPGs are superior to the mould-casted specimens in terms of compressive strength, ductility, flexural resistance, and shrinkage. It is shown that although the lamellar structure of 3D-printed samples has a potential weak interlocking surface, good mechanical properties can be obtained by 3D printing.

## 7. Improvement of Weak Interlayer Bonding

To solve the problem of weak interlayer bonding strength of 3DPGs, researchers have proposed a variety of optimization methods. In the preparation of the 3DPGs mixture, the materials that can improve the interlayer bonding property of printing can be added to the mixture. It is also possible to add a layer of materials with good bonding properties to the mix between the printing layers [100,101,102,103,104,105] or add modified materials to improve the bonding properties between the fibers and the matrix [106,107,108,109,110,111,112]. In addition, the fibers can also be pretreated, with reasonable fiber characteristics, to improve the construction process conditions and increase the performance of printing equipment so that the fiber penetrates the interlayer interface of slurry. In recent years, researchers have adopted a variety of measures to improve the interlayer bond strength during 3DPG printing through improvements in materials, equipment, techniques, and other factors [113,114,115,116].

### 7.1. Good Material Formulation

Printing material formulations is one of the main challenges of 3DFRPGs. A printing material with good properties must be able to pump out and, at the same time, retain its shape after extrusion so that the material has suitable printing properties. Weng et al. [117] studied the effect of the dosage of a highly effective water-reducing agents on the interlayer bonding strength of 3DPGs. By increasing the dosage of water reducing agent, the thixotropy index reduced, the surface water content was raised, and the interface microstructure and interlayer bond strength improved. Xia et al. [118] used FA powder to prepare printing mortar. The higher FA content increased the diffusion diameter of the interlayer binder but decreased the penetration depth of the binder. Szostak et al. [119] also improved the plasticity of the 3DPGs mixture by adding FA. At the same time, a readily available aqueous solution containing calcium silicate hydrate nanocrystals, namely C-S-H nanocrystals, was added. The use of a C-S-H nano-admixture could shorten the solidification time and increase the strength of the mixture quickly. Murcia et al. [67] designed high-performance 3DPGs by selecting high-content volcanic ash materials, considering rheology, plasticity, and constructability. The compressive strength increased obviously after hardening. Li et al. [95] added a viscosity modifier, which effectively eliminated the influence of interlayer stratification on the fracture path. Bong et al. [93,120] significantly improved the inter-layer tensile strength of printing by adjusting the ratio of printing materials. Panda et al. [121] developed a one-part geopolymer that can adjust its viscosity, control its setting and hardening rate, and extrude deposition layer by layer without affecting the flow characteristics of the mixture. Kong et al. [72] introduced and added kenaf rod and kenaf fiber, and the synergistic effect of the kenaf rod skeleton and fiber bridge kept the 3DFRPGs mixture in good shape. Weng et al. [85] evaluated the amount of 3DFRPGs fiber. The results show that the rheological parameters of FR3DPG increase with fiber content and decrease with the rise of the water-binder ratio. Ding et al. [97] believe that the post-loading peak performance is directly related to the fiber content, and selecting appropriate PE fiber length is the critical factor.

### 7.2. Improving the Printing Process

The interlayer bond is closely related to the nozzle specification, printing height, printing speed, and other parameters of the printing equipment. Weng [117] studied the influence of printing speed, curing conditions, and other parameters on the interlayer bonding strength of 3DPGs at different stages. By increasing the pumping speed to increase the shear rate of the material, the interlayer bonding strength was improved. At the same time, appropriate curing could also improve the interlayer bonding strength. Ma et al. [69] mechanically strengthened the fiber arrangement along the printing direction by keeping the nozzle diameter smaller than the length of basalt fibers. Zareiyan et al. [93] improved the interlayer splitting strength by reducing the printing strip height. Panda et al. [122] improved the inter-layer tensile strength of 3DPGs by adjusting the running speed and printing height of the nozzle. The experimental results show that the interlayer direct tensile strength of the specimen with a 70 mm/s nozzle running speed is 10.5% higher than that with a 110 mm/s nozzle running speed. The direct interlayer tensile strength of the specimen with a 0 mm nozzle height was 53.3% higher than that with a 4 mm nozzle height. Alghamdi [123] discussed the printing parameters of extruded base 3DPGs, including nozzle shape and printing speed. Xia et al. [118] put forward post-processing methods for improving green strength. Post-treatment significantly enhances the strength of the material. Post-processing specimen printed with GGBS (50 wt%) and FA (50 wt%) seven days compressive strength can be up to 25 MPa. The parameters comparison of the different printing processes can be seen in Table 4. Printer nozzles include rectangles and circles. Nozzle shape and flow type (back flow, down flow, hybrid flow) will affect the bond between layers. In the table, all printer nozzles are rectangular. Generally, different printing processes can be selected according to different materials and conditions. In most of these studies, the interlaminar bond strength is measured by the uniaxial tensile method, and the splitting tensile strength can also be used.

### 7.3. Improving Compactness of Layer Interface

Generally, the compactness of the printing layer interface has a significant influence on the interlayer bond strength. Kong et al. [72] designed six printing nozzles with different geometries to optimize interlayer bonding of multilayer printed specimens of different shapes and sizes. Yalçınkaya et al. [68] found that the local porosity between layers was closely related to the surface roughness after extrusion by printing three layers of steel fiber-reinforced cement-based specimens. Beushausen et al. [126] constructed gaps of different degrees at the interface to improve the bonding strength of the old and new interfaces. Zareiyan et al. [93] tested other interlocking structures and showed that the bonding strength was sensitive to interlocking, and the splitting test showed that it could be increased by 26% on average. Sun et al. [127] analyzed the bonding properties between basalt fibers and matrix by drawing tests. The results showed that the bonding properties of sand-coated fibers were better than those of smooth. The printing direction affected the bond performance of 3DPGs, and the bond strength of parallel and 45-degree inclined specimens exceeded that of vertically printed specimens. Ma et al. [30] created additional mechanical bonding by interlocking fibers and fine aggregates. The interlaminar tensile strength increased to more than 1.91 MPa in the 60 min printing time interval. This also improves the filament deposition and stacking process, reducing voids and longitudinal defects and enhancing durability.

Usually, the printing outage time is too long for the structural, mechanical properties, and durability of the negative impact. In this case, it is essential to apply high-efficiency binders and interface intensifies between printing layers to reduce the influence of weak surfaces [128].

### 7.4. Adjustment of Printing Interval

Reasonable printing process parameters are helpful for improving the interlayer bonding strength of 3DFRPGs, and the printing time interval has a prominent effect on it. Panda et al. [129] found that the larger the time interval between layers, the lower the intensity, and the lower the printing speed and nozzle distance, the better the effect. The printer parameters can be optimized according to the printing material type, specimen size and shape to improve process performance. Sakka et al. [130] used polymer film to better combine hydration products, effectively reducing the adverse impact of interval time on the interlaminar tensile strength of printed specimens. When the interval extended to 15 min, the interlaminar axial tensile strength of the printed specimen decreased by only 3.5%. The experimental results of Wolfs et al. [90] showed that reducing the interlayer interval time could improve the interlayer tensile strength. The interlaminar tensile strength and split tensile strength of specimens with an interval of 15 s were 19% and 27% higher than those with an interval of 24 h, respectively. In addition, the interlaminar tensile strength can improve by adequately covering the specimens. The experimental results showed that the interlaminar tensile strength of the specimens covered with 4 h intervals was 102% and 36% higher than that of the exposed specimens with 4 h and 24 h interval, respectively. The shorter the interlayer interval time, the more the surface coverage improves the interlayer tensile strength. Le et al. [81] designed a printer with a 9 mm diameter nozzle and used an extrusion process to print a high-performance fiber-reinforced mixture. The interlaminar tensile bonding strength was 2.3–0.7 MPa, which decreased with the increase in printing time interval. The well-printed mixture had significantly fewer voids with diameters greater than 0.2 mm (1.0%), while the poorly printed combination had more voids (4.8%). Tay et al. [131] studied the influence of time interval on the printing filament through rheological and macroscopic scale observation. They showed that the tensile strength of the printed specimen was related to the material modulus of the initial layer. The novel 3DFRPGs proposed by Ma et al. [101] could prolong the printing time interval and deal with the problem of filament breakage.

### 7.5. Optimization of Printing Path

In addition to the time interval, the printing path is also one of the main factors affecting the bonding strength between 3DFRPGs layers. Ma et al. [49] also printed 3DFRPGs specimens with three different printing path configurations and studied different printing paths. Al-Qutaifi et al. [27] studied 18 different printing specimens. The specimens included three different fiber contents, three different printing time intervals of 5, 10, and 15 min, and two layering modes. The addition of fibers during the delamination process affects the bond strength between subsequent layers. Sun et al. [131] found that under the condition that the extrusion shape and size were unchanged, the staggered arrangement of printing ports could improve the mechanical properties of printing specimens by 13–47%. The interlayer and inter strip defects could significantly affect the compactness of the printed specimens. Under the same extrusion flow rate, the printed specimens with triangular extrusion shape had the most negligible interlayer defects and the best mechanical properties due to the compression between the layers and strips. With the increase in extrusion size, the flaws of printed specimens with the same shape decreased, and the mechanical properties improved.

## 8. Conclusions

The fiber reduces the fluidity of 3DFRPGs and has little effect on the compressive resistance of 3DFRPGs, but the flexural resistance of 3DFRPGs is significantly enhanced. The flow of the mixture, printing direction, and printing interval significantly affect the performance of 3DFRPGs. Based on this study, the following conclusions can be drawn for 3DFRPGs.

3DFRPGs are formed by layer-by-layer stacking, which tends to cause weak bonding between layers, resulting in anisotropic behavior. The diversity and environmental protection of plant fibers and the excellent properties of polymer fibers should be fully utilized.

Compared with plant fibers, inorganic fibers showed a marked lack of sticking to the matrix of geopolymers. The mechanical properties of carbon fiber-reinforced composites are inferior to those of flax fiber-reinforced composites. Using steel fibers prevents complete adhesion between the print layers.

However, good mechanical properties can still be obtained by 3D printing. Both flexural strength and compressive strength are affected by loading direction. Compared with the specimens loaded perpendicular to the printing direction, the flexural strength of the specimens loaded perpendicular to the printing layer increased by 110% and 43% at 7 and 28 days, respectively.

In addition, according to the actual engineering situation, the interlayer bond performance of 3DFRPGs can be improved by adding functional materials, improving the printing process, shortening the printing interval and other effective methods.

## Figures and Tables

**Table 1:** The filler materials and their related analysis as additive materials of 3DPGs [27,41,42,43,44,45,46,47,48,49,50].

Fiber	Fiber Content /%	Type of Binder	Binder	Fine Aggregate/%	Additive /%	Ref.
polypropylene	0.25–1.00	GP	100 (FA)	150.00	2–0.40(CMC)	[41]
	0.25	GP	100 (FA)	108.70	0.24(CMC)	[42]
	1.00, 1.50, 2.00	OPC + GP	18 (FA), 38 (SF)	92.07	-	[43]
	0.56	GP	64 (FA), 25 (GGBS), 11 (SF)	120.00	(HEC)	[44]
	0.5	GP	100 (FA)	100.00	-	[27]
polyvinyl alcohol	0.25	GP	100 (FA)	108.70	0.52(CMC)	[42]
	2.00	GP	50 (FA), 50 (GGBS)	100.00	0.20(CMC)	[45]
	1.75	OPC + GP	55 (GGBS), 5(SF)	36.00	0.6 (SP)	[46]
Polyphenylene benzoxazole	0.25	GP	100 (FA)	108.70	0.10(CMC)	[42]
Steel	1.00	GP	100 (FA)	100.00	-	[27]
Glass	0.25, 0.5, 0.75, 1.00	OPC + GP	23 (FA),5 (GGBS), 3 (SF)	47.00	2.00(HPMC)	[47]
Carbon	1.00	GP	100 (FA)	100.00	-	[48]
Basalt	0.10, 0.30, 0.50, 0.70	OPC + GP	29 (FA), 14 (SF)	171.00		[49]
Flax	1.00	GP	100 (FA)	100.00	-	[48]
Sisal	0.60, 1.00	OPC + GP	22(FA), 12(MTK)	188.00	0.60 (SP)	[50]

In the table, GP-geopolymer, OPC-Portland cement, MTK-metakaolin. CMC-Sodium carboxymethyl cellulose, HEC-Hydroxyethyl cellulose, HPMC-Hydroxypropyl Methyl cellulose, SP-superplasticiser.

**Table 2:** Properties of common fibers of 3DFRPGs [41,42,48,50,52,53,54,55,56,57,58].

Fiber Type	Fiber Name	Density /g/cm^3^	Tensile Strength/MPa	Specific Strength	Tensile Modulus/GPa	Specific Modulus	Elongation at Break/%	Ref.
Synthetic organic	Polypropylene	0.90	880	978	13.2	14.7	17.6	[41]
	Polyvinyl alcohol	1.30	1600	1231	37	28.5	6.0	[42]
	Polyphenylene benzoxazole	1.56	5800	3718	270	173.1	2.5	[42]
Inorganic	Carbon	1.60	5000	3125	230	143.8	1–1.5	[48]
	SUS304	7.90	515	65	193	24.4	40	[52]
	Steel	7.80	800	103	200	25.6	1.2	[53]
	Basalt	2.55	2180	855	87.2	34.2	2.55	[50]
Plant	Flax	1.50	800–1500	535–1000	27.6–80	18.4–53	1.2–3.2	[54]
	Hemp	1.48	550–900	372–608	70	47.3	2–4	[55]
	Jute	1.46	393–800	269–548	10–30	6.85–20.6	1.5–1.8	[56]
	Kenaf	1.45	930	641	53	36.55	1.6	[57]
	Sisal	1.45	530–640	366–441	9.4–22	6.5–15.2	3–7	[41]
	Cotton	1.60	287–597	179–373	5.5–12.6	3.44–7.9	7–8	[58]
	Coir	1.20	175	146	4–6	3.3–5	30	[41]
	Wood	1.50	1000	667	40	26.67	4.4	[58]

**Table 3:** Main components and property measurements of 3DFRPGs in some studies [27,44,47,52,55,59,60,61].

Binder/%			Activator	A/B	Fiber Content /%	Aspect Ratio	Property Measurement	Ref.
FA	GGBS	SF						
80	15	5	K_2_SiO_3_	0.45	0.5 (PVA)	5714	Thixotropic, flexural, microstructure	[52]
50	50	-	Na_2_SiO_3_ (Anhydrous)	0.40	1.0 (PVA)	231	Thixotropic, deformation, stiffness, interlayer strength, microstructure	[59]
74	16	10	K_2_SiO_3_	0.65	0.25–1.0 (Glass)	3, 6, 8 ^a^	Compressive, flexural, and tensile strength	[47]
100	-	-	NaOH + Na_2_SiO_3_ (D grade)	0.52	0.5 (PP)	227	Buildability, flexural strength	[27]
					1.0 (Steel)	65		
64	25	11	Na_2_SiO_3_⋅5H_2_O	0.31	0.8 (Stainless steel cable)	1200 ^b^	Flexural behavior	[44]
64	25	11	Na_2_SiO_3_⋅5H_2_O	0.31	0.8 (Stainless steel cable)	1200 ^b^	Compressive, tensile, shear, and pull-out behavior	[60]
50	50	-	Na_2_SiO_3_ (Anhydrous)	0.28	2.0 (PVA)	200	Density, porosity, compressive, and flexural strength	[55]
87.7	4.6	7.7	NaOH + K_2_SiO_3_	0.49	0.25 (Glass)	4 ^a^	Static yield stress, thixotropic, shape retention ability, deformation	[61]
83.1	9.2	7.7						
78.5	13.8	7.7						

^a^ is the length of fibers, measured in mm. ^b^ is the fiber diameter, measured in μm; A/B is the quality ratio of Activator and Binder.

**Table 4:** Parameters of the different printing processes [41,46,47,52,87,92,117,121,122,123,124,125].

Author	Nozzle Dimension (mm)	Printer Speed (mm/s)	Layer Height (mm)	Layer Width (mm)	Time Interval (min)	Curing Conditions	Ref.
Nematollahi et al.	15 × 25	-	15	25	15	Ambient	[41]
Aslani et al.	20	10–100	-	-	-	Standard moist	[46]
Panda et al.	40 × 10	-	10	40	0.13	laboratory temperature	[47]
Lim et al.	13 × 30	60	13	30	-	Ambient	[52]
Sanjayan et al.	15 × 25	12	25	15	10, 20, 30	Moist and temperature (23 ± 3 °C)	[87]
Chaves et al.	10 × 50	100	10	50	3, 2, 4.5	(20 ± 2)°C and relative humidity of (98 ± 2)%	[92]
Panda et al.	40 × 10	90		30	1	Ambient	[121]
Weng et al.	15 × 30	14.5–66.7	15	30	-	Air; Water; Standard	[117]
Panda et al.	30 × 15/20 × 20	70–110	20	20	1–360	Ambient	[122]
Tay et al.	30 × 15	80	15	30	1, 5, 10, 20	Temperature (23 ± 2 °C) and relative humidity at 60%.	[124]
Panda et al.	15 × 7	120	7	15	5–20	Ambient	[125]

## Data Availability

Not applicable.
